# Supplementary material for: Thermally activated irreversible homogenization of G-quadruplexes in an ALS/FTD-associated gene
Source: bioRxiv. 2025 Nov 24:2025.06.02.657482. Preprint. [Version 2] doi: 10.1101/2025.06.02.657482 (PMC12697674; doi:10.1101/2025.06.02.657482)
Supplement: 1 [file NIHPP2025.06.02.657482V2-supplement-1.pdf]

bioRxiv preprint doi: https://doi.org/10.1101/2025.06.02.657482; this version posted November 24, 2025. The copyright holder for this preprint (which was not certified by peer review) is the author/funder, who has granted bioRxiv a license to display the preprint in perpetuity. It is made available under aCC-BY 4.0 International license.

SUPPLEMENTARY INFORMATION FOR "THERMALLY ACTIVATED IRREVERSIBLE HOMOGENIZATION OF G-QUADRUPLEXES IN AN ALS/FTD-ASSOCIATED GENE"

Daniel Ross[1,*], Olivia Lewis[1], Olivia McLean[1], Sundeep Bhanot[1], Shane Donahue[1], Rachael Baker[1], Randi Dias[1], David Eagerton[1], Vaibhav Mohanty[2,3,4,*], Bidyut K. Mohanty[1,*]

[1]Edward Via College of Osteopathic Medicine–Carolinas Campus, Spartanburg, SC 29303
[2]Department of Chemistry and Chemical Biology, Harvard University, Cambridge, MA 02138
[3]Harvard/MIT MD-PhD Program, Harvard Medical School, Boston, MA 02115 and Massachusetts Institute of Technology, Cambridge, MA 02139
[4]Program in Health Sciences and Technology, Harvard Medical School, Boston, MA 02115 and Massachusetts Institute of Technology, Cambridge, MA 02139
[*]Correspondence: dross@vcom.edu (D.R.); mohanty@hms.harvard.edu (V.M.); bmohanty@vcom.edu (B.K.M.).

Supplementary Note A MODULATING p_{est} PERFORMS AN AFFINE SHIFT ON $p(\tau, T_i)$

Suppose the experimental CD spectrum at 20 °C, $\epsilon_{\text{mix}}(\lambda)$ is given by a linear combination of parallel and antiparallel basis spectra, $\epsilon_P(\lambda)$ and $\epsilon_A(\lambda)$, respectively, with a guessed PG4 fraction p_{est} and NPG4 fraction $1 - p_{\text{est}}$:

$$\epsilon_{\text{mix}}(\lambda) = p_{\text{est}}\epsilon_P(\lambda) + (1 - p_{\text{est}})\epsilon_A(\lambda). \tag{A1}$$

The estimated antiparallel basis spectrum is solved in terms of the experimentally observed room temperature (mixed) spectrum $\epsilon_{\text{mix}}(\lambda)$ and the presumed parallel spectrum (obtained at high temperature) $\epsilon_P(\lambda)$:

$$\epsilon_A(\lambda) = \frac{\epsilon_{\text{mix}}(\lambda) - p_{\text{est}}\epsilon_P(\lambda)}{1 - p_{\text{est}}} \tag{A2}$$

The theoretical 3D spectrum $\epsilon_{3\text{D}}(\lambda, T_i)$ is given by main text eq. (6). Substituting the above equation for $\epsilon_A(\lambda)$, we have

$$\begin{aligned} \epsilon_{3\text{D}}(\lambda, T_i) &= p(\tau; T_i)\epsilon_P(\lambda) + (1 - p(\tau; T_i))\left[\frac{\epsilon_{\text{mix}}(\lambda) - p_{\text{est}}\epsilon_P(\lambda)}{1 - p_{\text{est}}}\right] \\ &= \frac{p(\tau; T_i) - p_{\text{est}}}{1 - p_{\text{est}}}\epsilon_P(\lambda) + \frac{1 - p(\tau; T_i)}{1 - p_{\text{est}}}\epsilon_{\text{mix}}(\lambda). \end{aligned} \tag{A3}$$

Letting

$$\alpha(T_i) \equiv \frac{p(\tau; T_i) - p_{\text{est}}}{1 - p_{\text{est}}}, \tag{A4}$$

we can now write

$$\epsilon_{3\text{D}}(\lambda, T_i) = \alpha(T_i)\epsilon_P(\lambda) + (1 - \alpha(T_i))\epsilon_{\text{mix}}(\lambda). \tag{A5}$$

Thus, the 3D CD spectrum is only affected by changes to $\alpha(T_i)$, which is a linear function of the $p(\tau; T_i)$ given by the thermodynamic-kinetic theory in the main text. If we choose a different p_{est}, it simply affects via an affine shift of the curve $p(\tau; T_i)$.

bioRxiv preprint doi: https://doi.org/10.1101/2025.06.02.657482; this version posted November 24, 2025. The copyright holder for this preprint (which was not certified by peer review) is the author/funder, who has granted bioRxiv a license to display the preprint in perpetuity. It is made available under aCC-BY 4.0 International license.

SUPPLEMENTARY TABLE

Number of Repeats	Replicate Number	T_{peak} (°C)	Figure	Additional conditions
2	1	81.98	S2	
4	1	86	2, repeated in S2	
4	2	94.02	S4	
4	3	91.98	S4	300 second wait time
6	1	81.99	S2	
6	2	79.99	S3	
10	1	88.02	S2	
14	1	84.02	2, repeated in S2	
14	2	84	S3	
16	1	84.02	S2	
16	2	83.99	S5	300 second wait time
16	3	84	2	with PEG, extended temperature range
17	1	82.02	S2	
18	1	84.01	S2	
20	1	83.98	2, repeated in S2	

Table S1: Summary of all 3D CD spectra obtained, their T_{peak} values, and their locations within this paper.

bioRxiv preprint doi: https://doi.org/10.1101/2025.06.02.657482; this version posted November 24, 2025. The copyright holder for this preprint (which was not certified by peer review) is the author/funder, who has granted bioRxiv a license to display the preprint in perpetuity. It is made available under aCC-BY 4.0 International license.

SUPPLEMENTARY FIGURES

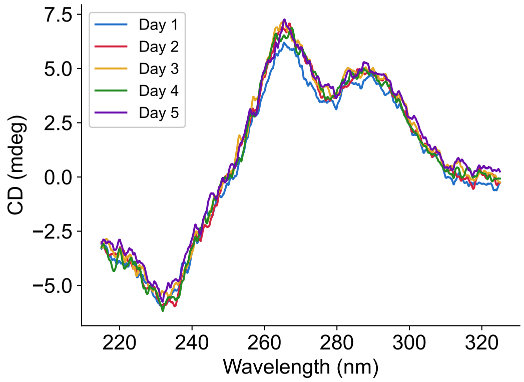

Figure S1: For 17 repeats of GGGGCC, the CD spectrum remained the same and stable at room temperature each day, for 5 days.

bioRxiv preprint doi: https://doi.org/10.1101/2025.06.02.657482; this version posted November 24, 2025. The copyright holder for this preprint (which was not certified by peer review) is the author/funder, who has granted bioRxiv a license to display the preprint in perpetuity. It is made available under aCC-BY 4.0 International license.

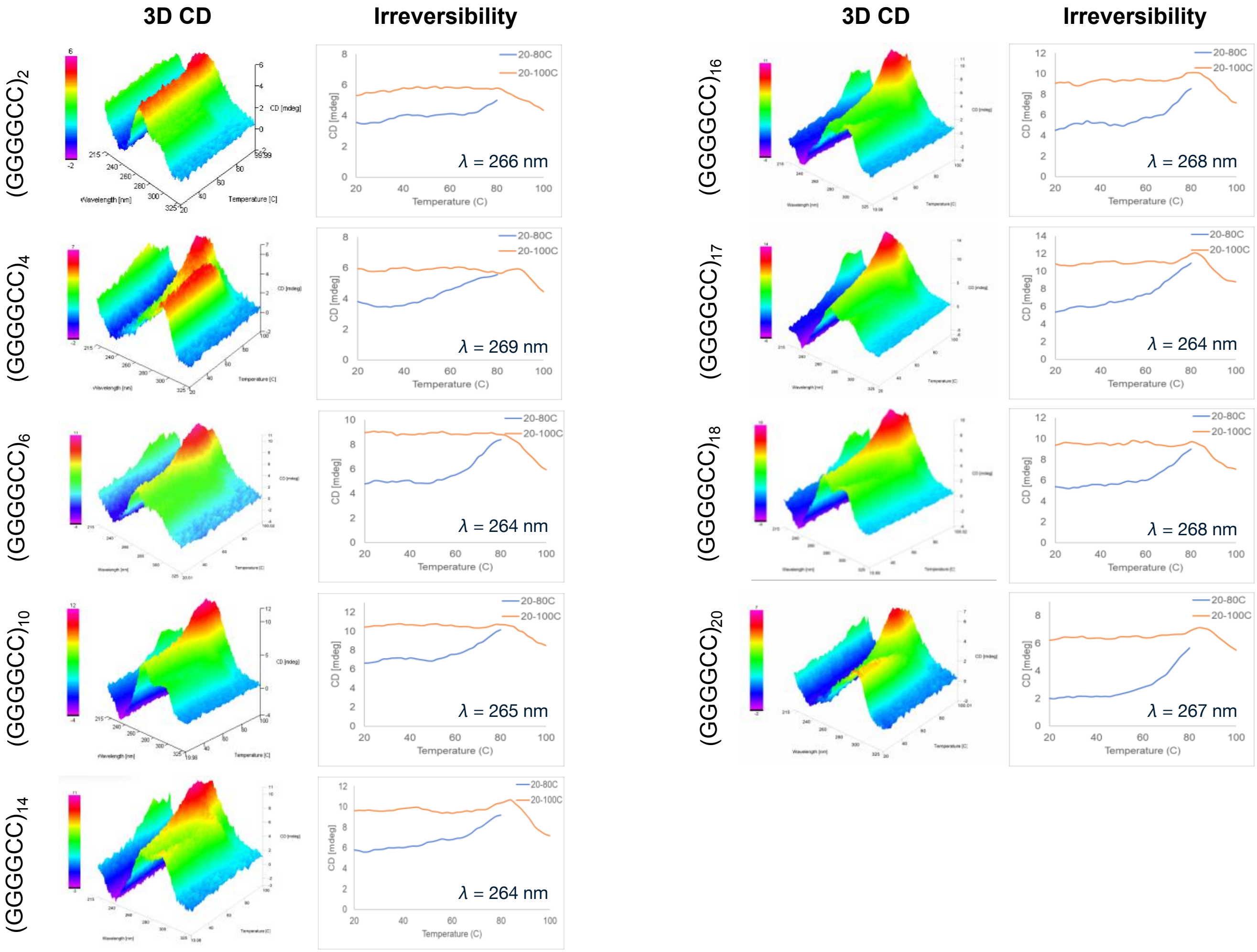

Figure S2: **3D CD spectra and irreversibility data for additional oligonucleotides with varying hexanucleotide repeat copy numbers.** 3D CD spectra demonstrate the metastable-to-stable homogenization transition consistent with theory, and consistent across hexanucleotide repeat copy numbers. Irreversibility data show stability of the CD peak during the second temperature sweep (20 °C to 100 °C, orange) after it was reached at the end of the first temperature sweep (20 °C to 80 °C, blue). Irreversibility data are consistent across hexanucleotide repeat copy numbers. The wavelengths used to capture irreversibility data for each copy number are listed and are all in the 264-269 nm.

bioRxiv preprint doi: https://doi.org/10.1101/2025.06.02.657482; this version posted November 24, 2025. The copyright holder for this preprint (which was not certified by peer review) is the author/funder, who has granted bioRxiv a license to display the preprint in perpetuity. It is made available under aCC-BY 4.0 International license.

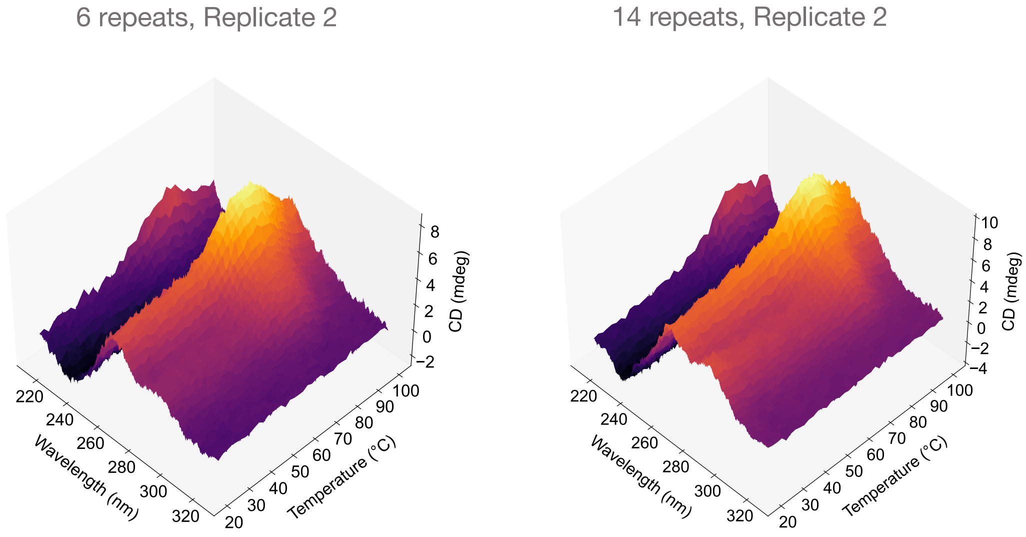

Figure S3: Additional experimental replicates for 6 and 14 repeats not utilized in other figures. Results are qualitatively similar to Figure S2.

bioRxiv preprint doi: https://doi.org/10.1101/2025.06.02.657482; this version posted November 24, 2025. The copyright holder for this preprint (which was not certified by peer review) is the author/funder, who has granted bioRxiv a license to display the preprint in perpetuity. It is made available under aCC-BY 4.0 International license.

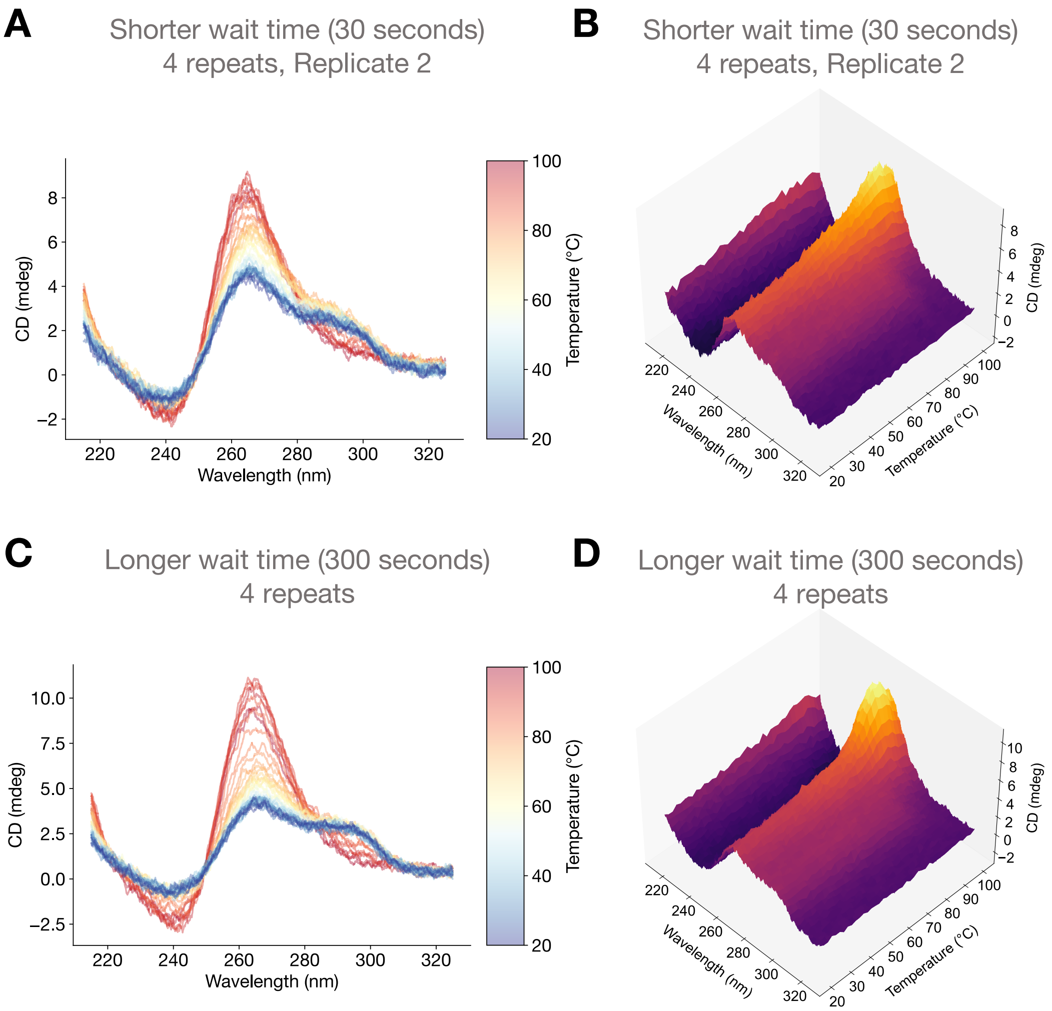

Figure S4: For 4 repeats of GGGGCC, we plot the temperature sweep experiments with (**A**,**C**) color-coded 2D and (**B**,**D**) 3D spectra with both shorter (30 second) and longer (300 second) wait times between each 2 °C increment.

bioRxiv preprint doi: https://doi.org/10.1101/2025.06.02.657482; this version posted November 24, 2025. The copyright holder for this preprint (which was not certified by peer review) is the author/funder, who has granted bioRxiv a license to display the preprint in perpetuity. It is made available under aCC-BY 4.0 International license.

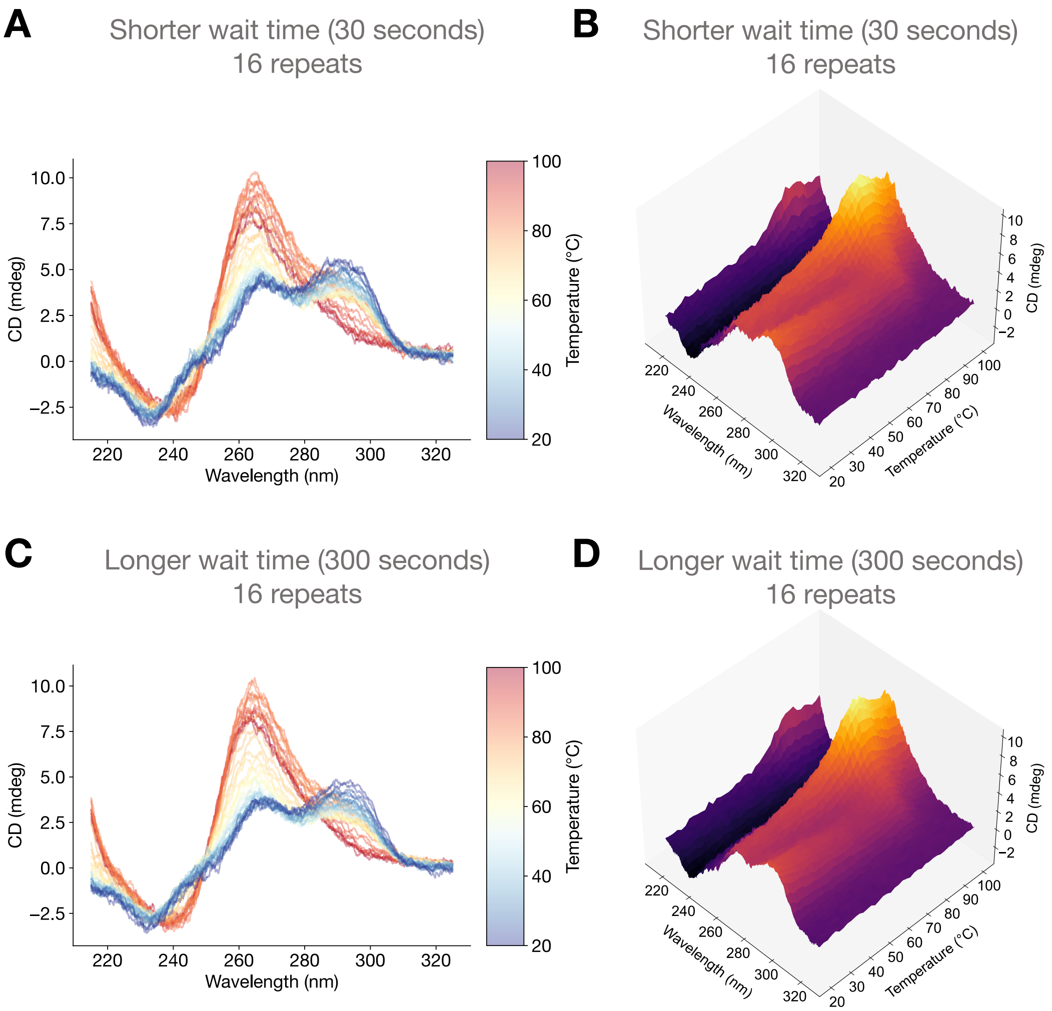

Figure S5: For 16 repeats of GGGGCC, the temperature sweep experiments yielded similar (**A**,**C**) color-coded 2D and (**B**,**D**) 3D spectra with both shorter (30 second) and longer (300 second) wait times between each 2 °C increment.

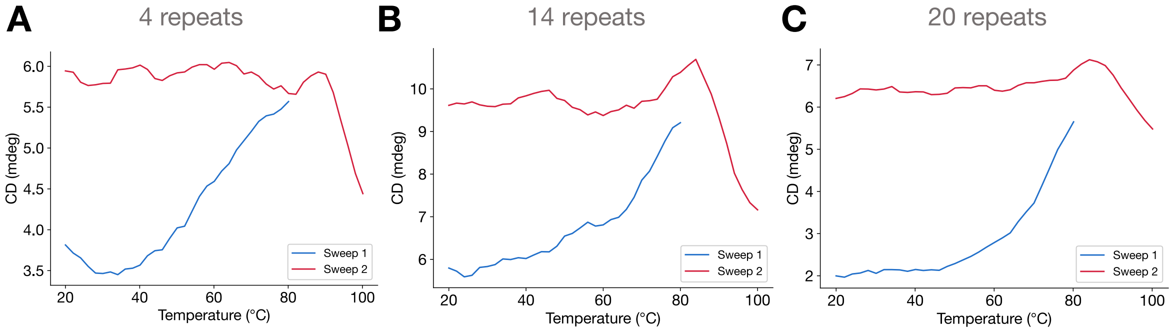

Figure S6: **Thermally activated metastable-to-stable G4 transition is irreversible**. (**A**) For 4 hexanucleotide repeats, the first temperature sweep (blue) shows a CD increase at 269 nm as temperature increases from 20 °C up to 80 °C, indicative of a transition to PG4s. After cooling back to 20 °C, the second temperature sweep (red) shows stability of the CD peak from 20 °C up to roughly 80 °C, after which CD decreases, either due to G4 melting (or possibly NPG4 formation). (**B**) Same as (A), but with 14 hexanucleotide repeats with CD measured at 264 nm. (**C**) Same as (A) and (B), but with 20 hexanucleotide repeats with CD measured at 267 nm. CD lines were smoothed with a moving average filter using a convolution kernel with a 5-datapoint width.
